# Reusable tourniquets for blood sampling as a source of multi-resistant organisms– a systematic review

**DOI:** 10.3389/fpubh.2023.1258692

**Published:** 2023-11-13

**Authors:** Julia Szymczyk, Michelle Månsson, Wioletta Mędrzycka-Dąbrowska

**Affiliations:** ^1^Student Scientific Club of Anesthesia and Intensive Care, Medical University of Gdańsk, Gdańsk, Poland; ^2^Student Nursing Programme, Swedish Red Cross University, Huddinge, Sweden; ^3^Department of Anesthesiology Nursing & Intensive Care, Faculty of Health Sciences, Medical University of Gdańsk, Gdańsk, Poland

**Keywords:** tourniquet, cross infection, nosocomial infection, *Staphylococcus aureus*, MRO, pathogen, infectious disease

## Abstract

**Introduction:**

The use of reusable tourniquets is widespread around the world, and reports suggest they may be overused. Several studies have shown that reusable tourniquets can affect the spread of pathogens between patients. Based on available studies, this review aims to analyse the indirect transmission of antimicrobial-resistant pathogens present on blood collection tourniquets, which may spread infectious diseases between patients in daily clinical practice.

**Methods:**

A systematic review of the literature was conducted according to the PRISMA (Preferred Reporting Items for Systematic Reviews and Meta-Analysis) protocol guidelines. The contents of PubMed, EBSCO (electronic databases), and Scopus were screened. Keywords used in the search included: “tourniquet,” “cross infection,” “nosocomial infection,” “*staphylococcus aureus*,” “MRO,” “pathogen,” “infectious disease,” “anti-microbial,” or a combination of these using AND or OR operators. Finally, 13 publications were included. Data were analysed both descriptively and quantitatively by calculating a balanced average for specific synthesized data.

**Results:**

The proportional observation based on the number sampled median was 77. The genus MRSA was the type of bacteria most commonly found: on 12% of all tested tourniquets. The amount of MRSA found on tourniquets was mean ± SD 14.6 ± 45.89. A review of studies also revealed the presence of *coagulase-negative staphylococci*, grew *Bacillus,* and *Staphylococcus aureus*.

**Conclusion:**

Patient safety may be at risk due to elevated contamination rates of reusable tourniquets. The microorganisms responsible for this contamination include a variety of species, the most common being the genus *Staphylococcus*. For this reason, we recommend the use of disposable tourniquets.

## Introduction

Collecting blood through peripheral venous access using tourniquets is one of the most common invasive procedures in hospitals and other medical facilities. However, tourniquets for venipuncture are non-sterile and potentially reusable equipment. The usual technique for providing venous access is to apply a reusable tourniquet to the patient’s arm (1). Conventional tourniquets are made of fabric, and their porous structure can be a potential reservoir of infection. Usual tourniquets can be recycled through sterilization or dipping in disinfectants. Such methods are time-consuming and unsuitable for immediate use between patients (2). The most common way to disinfect reusable tourniquets is to spray them with disinfectant and hang the tourniquet to dry, but such a disinfection method is not effective as it is time-consuming. The use of such tourniquets, transferred between multiple patients, contributes to the transmission of multiresistant microorganisms (MROs) between patients and contradicts basic infection control principles (3–6). Reusable tourniquets used for blood collection are a potential carrier of multiresistant microorganisms. Disinfection of reusable equipment is not clear-cut, thus the use of disposable tourniquets, which do not require disinfection and are disposable, is preferable to reusable tourniquets. Several studies have shown that the use of reusable tourniquets can contribute to the transmission of MRO between patients. This is particularly important for immunocompromised patients who are at risk for nosocomial infections (2, 7). Improving the quality of health care involves increasing the quality and safety of medical devices used by health care workers, such as during blood draws (1). Since a review of the literature can identify major epidemiological and clinical findings, our purpose was to provide a comprehensive report on the proportion of sampled specimens between the use of reusable tourniquets and the pathogens detected on them based on recent studies.

## Methods

### Study design

The literature review was performed in the second quarter of 2023. This systematic review was carried out in accordance with the PRISMA (Preferred Reporting Items for Systematic Reviews and Meta-Analyses) statement using PICO-based questions (patients, interventions, comparisons, and outcomes). The resources of PubMed, EBSCO (electronic databases), Scopus, and MEDLINE were searched. No filters were used during the search, and the search included backward citation of eligible full-text trials.

### Evidence acquisition

In May 2023, three independent researchers (JS, MM, and WM-D) conducted a search of the targeted online databases for eligible studies. The keywords used were “tourniquet,” “cross-infection,” “nosocomial infection,” “*staphylococcus aureus*,” “MRO,” “pathogen,” “infectious disease,” “anti-microbial” or a combination of these using AND or OR operators. The preliminary search returned 291 articles, 13 of which were included in further analysis. Papers published in English were included in the analyses.

### Inclusion and exclusion criteria

The inclusion and exclusion criteria were based on the PICOS classification. If articles fulfilled predefined criteria, they were included and classified as basic research (animal and cell studies), epidemiological (morbidity and mortality studies), and treatment studies. Articles were excluded if the full text was not publicly available was not available in English and if articles were not original articles or did not support PICO.

Inclusion criteria:

- articles in English
- research group consisting only of reusable tourniquets
- studies describing pathogens on reusable tourniquets

Exclusion criteria:

- articles in a language other than English or Polish
- research where a study group consisted of medical equipment different than reusable tourniquets

### Evidence synthesis and quality assessment

Three independent researchers (JS, MM, and WM-D) retrieved and summarized information from the eligible studies in tables. The authors discussed conflicts regarding study inclusion and managed to resolve them by consensus. Studies were considered to be of high quality with a tool created by the Joanna Briggs Institute (JBI) (8). The system created by the JBI was designed to provide reviewers with a comprehensive guide on how to conduct a systematic review and how to rank selected articles (JBL for manual synthesis)^1^. Additionally, an appraisal checklist with 11 criteria (Q1-Q11) was also used. The questions on the checklist centered on the inclusion criteria of the selected articles, the sources and resources of the selected material, and the type of approaches used in the study. The answers used were yes, no, unclear, or not applicable. The outcomes of the quality assessment are described in Table 1.

**Table 1:** Critical appraisal results for included studies.

Author, year	Q1	Q2	Q3	Q4	Q5	Q6	Q7	Q8	Q9	Q10	Q11
Elhassan H. A. et al. (2012) (9)	Y	Y	Y	Y	Y	Y	n/a	Y	Y	Y	Y
Mehmood Z. et al. (2014) (10)	Y	Y	Y	Y	Y	Y	U	Y	U	Y	Y
Leitch A. et al. (2006) (11)	Y	Y	Y	Y	N	Y	Y	Y	U	Y	Y
Golder M. et al. (2000) (3)	Y	Y	Y	Y	Y	Y	U	Y	N	Y	Y
Rourke C. et al. (2001) (12)	Y	Y	Y	Y	Y	Y	n/a	Y	Y	Y	U
Berman D. S. et al. (1986) (4)	Y	Y	Y	Y	Y	Y	n/a	Y	Y	Y	Y
Culjak M. et al. (2018) (5)	Y	Y	Y	Y	Y	Y	n/a	Y	N	Y	Y
Pinto A. N. et al. (2011) (13)	Y	Y	Y	Y	Y	Y	n/a	Y	U	Y	Y
Hensley D. M. et al. (2010) (6)	Y	Y	Y	Y	Y	Y	n/a	Y	U	Y	Y
Abeywickrama T. et al. (2010) (14)	Y	n/a	Y	Y	Y	Y	Y	Y	N	N	Y
Frankiln G. F. et al. (2017) (15)	Y	Y	Y	Y	Y	U	U	Y	Y	Y	Y
de Oliveira Batista K. C. et al. (2015) (16)	Y	Y	Y	Y	n/a	n/a	U	Y	N	Y	Y
Kane L. et al. (2011) (17)	Y	Y	N	n/a	U	n/a	n/a	Y	U	Y	Y

Y, yes; N, no; U, unclear; n/a, not applicable. Q1: Was the review question clearly and explicitly stated? Q2: Were the inclusion criteria appropriate for the review question? Q3: Was the search strategy appropriate? Q4: Were the sources and resources used to search for studies adequate? Q5: Were the criteria for appraising studies appropriate? Q6: Was the critical appraisal independently conducted by two or more reviewers? Q7: Were there methods to minimize errors in data extraction? Q8: Were the methods used to combine studies appropriate? Q9: Was the likelihood of publication bias assessed? Q10: Were recommendations for policy and/or practice supported by the reported data? Q11: Were the specific directives for new research appropriate?

## Results

**Figure 1:**
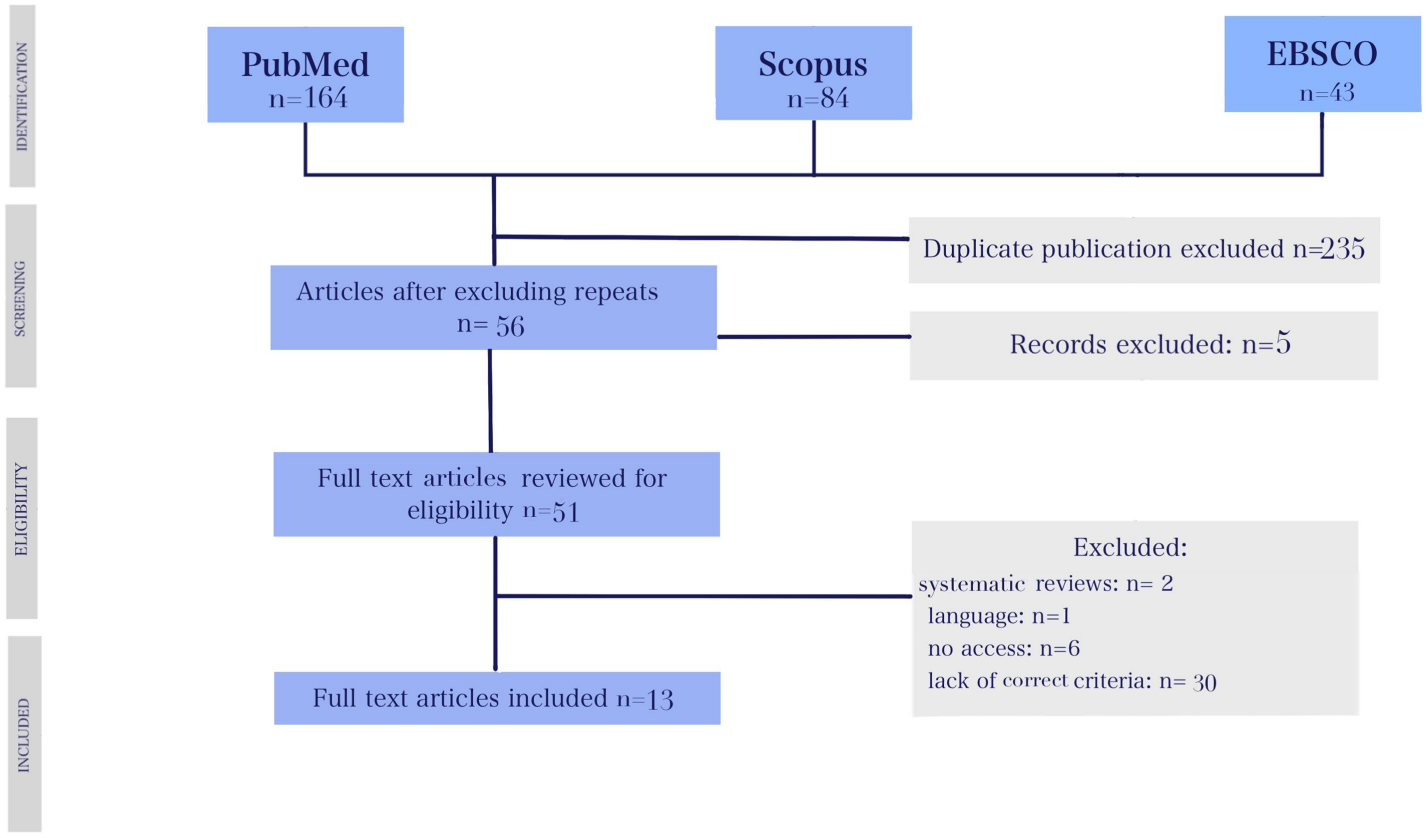
PRISMA flow diagram.

A total of 291 articles were found in the scientific databases. After removing duplicates, 56 papers remained for analysis. Then, 51 full-text articles were retained after reviewing abstracts. The next step focused on inclusion and exclusion criteria (38 were rejected). At the stage of qualitative text analysis, four articles were rejected. Finally, 13 articles were accepted for systematic analysis (Figure 1).

Some limitations need to be highlighted, such as limited observations due to the number of samples taken and the fact that there was no methodological correlation with regard to nosocomial infections caused by contaminated tourniquets.

The included studies were conducted between 1986 and 2018 in countries such as the United Kingdom, the United States, Canada, and Pakistan. The proportional observation based on the number sampled median was 77. The genus MRSA was the type of bacteria most commonly found: on 12% of all tested tourniquets, with the median of MRSA amounting to 8.5%. The amount of MRSA found on tourniquets was mean ± SD 14.6 ± 45.89. A review of studies also revealed the presence of *coagulase-negative staphylococci*, grew *Bacillus,* and *Staphylococcus aureus*.

A synthesis of the qualitative results of the review of the literature on reusable compression tourniquets in the field of MRO is presented in Table 2.

**Table 2:** Synthesis of qualitative results of literature review relating to reusable tourniquets in the field of MRO.

Author and year of publication	Aim	Study group	Materials and methods	Results	Implications for nursing practice
Elhassan H. A. et al. (2012) (9)	MRSA-contaminated venipuncture tourniquets in clinical practice.	50 reusable tourniquets collected from two district hospitals in Essex, UK	- The bag with a tourniquet and 10 ml of Ringer’s solution was shaken vigorously to elute the organism from the tourniquet. - A sample of the ‘tourniquets derived solution’ was dropped by pipette (one drop – 50 μL) and spread by sterile disposable loop onto agar with 5% horse blood, nalidixic acid, colistin, and mannitol – salt agar plates and incubated at 37°C aerobically for 48 h.	- 18/50 (36%) tourniquets were positive with *Staphylococcus aureus* and of these 6/50 (12%) were methicyllin-resistant *Staphylococcus aureus, MRSA* positive - 10/50 (20%) grew *bacillus* - 30/50 (60%) were visibly blood stained	Common patient devices, including disposable tourniquets, should be cleaned every time they are used. If shared equipment is unable to be cleaned among patients, disposable items are preferred.
Mehmood Z. et al. (2014) (10)	Potential risk of cross-infection by tourniquets: a need for effective control practices in Pakistan.	100 reusable tourniquets collected from public (40) and private (60) hospitals in Karachi, Pakistan	- To obtain the samples, swab sticks moistened with sterilized saline were rotated over both sides of the tourniquet at the distal and proximal ends. - The samples were streaked onto basic blood agar and McConkey’s agar culture medium.	- Bacterial growth was found on 23/40 samples from public sector hospitals and on 28/60 from the private sector hospitals - MRSA was more prevalent in public than in the private sector hospitals (18,2% vs. 16,6%) - *Staphylococcus aureus* was found on 12 of 100 tourniquets.	Ceasing multiple-use tourniquets, using cost-effective, disposable tourniquets, and introducing proactive prevention methods such as effective hand hygiene are the only effective measures to prevent cross-infection. The use of disposable tourniquets should be the norm, as there is no reliable way to disinfect reusable tourniquets.
Leitch A. et al. (2006) (11)	Reducing the potential for phlebotomy tourniquets to act as a reservoir for methicillin-resistant *Staphylococcus aureus*	131 reusable tourniquets	- The tourniquets were collected into specimen bags, coded, and transported to the laboratory. - In the laboratory, the tourniquets were pressed onto blood agar and mannitol salt, then swabbed lengthwise by a sterile moist swab. - This was then used to inoculate 7% salt broth. - After 24 h of incubation at 37°C, the broth was inoculated in lines across an ISO-sensitest plate.	- The rate of contamination with MRSA was 32 of 131 (25%) tourniquets. - 12/131 (9%) *Staphylococcus aureus* - 73/131 (56%) *coagulase-negative staphylococci*	The process of decontaminating reusable medical devices such as tourniquets was inadequate according to the authors, and the use of disposable ones should be implemented. Taint rates, and consequently the potential risk, can be decreased if hand decontamination is carried out.
Golder M. et al. (2000) (3)	Potential risk of cross-infection during peripheral-venous access by contamination of tourniquets	77 reusable tourniquets collected from a London teaching hospital and two large district hospitals, UK	Group A: -50 tourniquets were examined for visible bloodstains. They were pressed three times onto the blood-agar plates, which were incubated at 37°C in air.	17/50 tourniquets had bacterial pathogens: MRSA (12), *Escherichia coli* (1), *Pseudomonas aeruginosa* (1), *Stenotrophomonas maltophilia* (1), *Enterococcus faecalis* (1)	In areas at increased risk of HIV-1 or hepatitis B virus infection, there is a potential risk of virus transmission from tourniquets to the patient through areas of damaged tissue, such as venous access and monitoring sites, open wounds, eczema, cuts, and abrasions. Hand washing between patients is highlighted as an important component of nosocomial infections. The authors suggest the need for the use of disposable tourniquets.
			Group B: −27 tourniquets with visible bloodstains (group B) were tested for HIV-1 RNA and HBsAg and also ....	HIV-1 RNA nor HBsAg was detected in any of the group B tourniquets.	
Rourke C. et al. (2001) (12)	Poor Hospital Infection Control Practice in Venipuncture and Use of Tourniquets	200 reusable tourniquets were obtained from a teaching hospital in Sheffield, UK	- An area of the tourniquet in contact with the patient’s skin was pressed once onto a blood agar plate. - The plates were examined after 18 h of aerobic incubation at 37°C.	- *Staphylococcus aureus* was isolated from 10 /200 (5%) - 75/200 (37,5%) had visible blood stains - 99,5% tourniquets had *coagulase-negative staphylococci* and *micrococci* - Of those tourniquets testing positive for *Staphylococcus aureus,* 30% had visible blood stains.	It seems that more work should be conducted among staff at the facility where the study was conducted to increase awareness and implementation of the infection control policies and the benefits of hand washing and glove use.
Berman DS. et al. (1986) (4)	Tourniquets and nosocomial methicillin-resistant *Staphylococcus aureus* infections	24 reusable tourniquets in use by the medical house staff and hospital phlebotomists	No data	- *Staphylococcus aureus* was found on 12/24 (50%) - 7/24 (29%) MRSA - 5/24 (21%) phage type 88	The writers suggest that tourniquets may be an important pathway of transmission of nosocomial pathogens, as they are transferred from arm to arm and ought to be changed or disinfected frequently.
Culjak M et al. (2018) (5)	Bacterial contamination of reusable venipuncture tourniquets in tertiary-care hospital	52 reusable tourniquets	- Samples were taken using soaked-in sterile saline swabs. - The entire surface of the tourniquets was swabbed, including the buckle. Tourniquets were left for further use, without interrupting usual ward routines.	- Coagulase-negative *Staphylococcus* spp. 46/52 (75%) - *Bacillus* spp. 5/52 (8%) - *Corynebacterium* spp. 1/52 (2%) - *Staphylococcus aureus* 1/52 (2%) - MRSA 2/52 (3%) - *Enterobacter* spp. 1/52 (2%) - *Enterococcus* spp. 1/52 (2%) - No bacterial growth 4/52 (6%)	Authors strongly advise the use of disposable tourniquets to avoid cross-contamination and the spread of microorganisms between patients. Moreover, the infection prevention nurse educated the hospital personnel on appropriate hand hygiene.
Pinto A. N. et al. (2011) (13)	Reusable venesection tourniquets: a potential source of hospital transmission of multiresistant organisms.	100 reusable tourniquets	- Tourniquets were collected and immediately placed into polyethylene specimen bags, labelled, and transferred to the laboratory. - They were immersed in an enrichment medium and incubated overnight. - Fluid from the broth was then subcultured onto a variety of agar media: hore-blood agar, MacConkey agar, and selective agar media for the detection of MRSA, VRE, and resistant gram-negative bacteria including ESBL- and MBL-producing organisms.	- *Bacillus* spp. 54/100 (54%) - *Enterobacteriaceae* 26/100 (26%) - VRE 19/100 (19%) - *Pseudomonas* spp. 18/100 (18%) - methicillin-resistant *S. aureus* 14/100 (14%) - *Coagulase-negative. Staphylococci* 13/100 (13%) - *Enterococcus* spp. 9/100 (9%) - *methicilin-sensitive S. aureus* 1/100 (1%) - *Extended-spectrum* *ß- lactamases* 1/100 (1%) - *Metallo-ß-lactames* 1/100 (1%)	With the present high incidence rates of MRO, continued use of reusable tourniquets may not be justified in the hospital setting.
Hensley D. M. et al. (2010) (6)	*Acinetobacter baumannii* and MRSA contamination on reusable phlebotomy tourniquets.	200 reusable tourniquets	100 reusable tourniquets were collected after being used for 1 day in the outpatient blood collection center and were cultured, and growth was screened for MRSA using colonial morphology, catalase, Staphaurex, and Oxacillin screening agar.	- *Acinetobacter baumanii* was isolated from 11/100 (11%) - *Methicillin-susceptible S. aureus* was isolated from 2/100 (2%)	Reusable tourniquets might serve as a potential reservoir of microbial pathogens.
			100 reusable tourniquets were collected during morning blood collection rounds on inpatient wards and methods of screening for MRO were the same as in the first group.	- *Acinetobacter baumanii* was isolated from 3/100 (3%) - *Methicillin-susceptible S. aureus* was isolated from 3/100 (3%) No MRSA was isolated.	
Abeywickrama T. et al. (2018) (14)	Methicillin-resistant *Staphylococcus aureus* contamination of phlebotomy tourniquets and faucets.	206 reusable tourniquets	Using aseptic techniques these tourniquets were incubated overnight at 37°C in brain heart infusion broth. 200 μL of broth was subcultured onto CGROMagar MRSA medium and processed according to the manufacturer’s instructions.	53/206 (25,7%) tourniquets grew MRSA.	Single-use (i.e., throw away after a single use) plastic tubing from new infusion sets was significantly less contaminated with MRSA than either reused plastic tourniquets or reused tourniquets intended for a single patient. General standard hand hygiene practices need to be built into the assessment of healthcare workers in hospitals to minimise MRSA cross-contamination.
Frankiln G. F. et al. (2017) (15)	Phlebotomy tourniquets and MRSA	50 reusable tourniquets	The tourniquets were taken to the laboratory in sterile bags and treated with a sterile technique. The plastic or metal clip was discarded and the entire band was dipped into 150 mL of brain heart infusion broth. After overnight incubation, 200 mL of broth was cultured on an Oxacillin Resistance Screening Agar Base (ORSAB) plate. The plate was incubated for 24 h and tested for the presence of dark blue colonies, presumed to be identified as MRSA.	33/50 (66%) tourniquets were soiled 4/50 (8%) tourniquets were visibly blood stained 5/50 (50%) tourniquets grew MRSA	This study confirms the presence of MRSA on reusable tourniquets and demonstrates the prolonged use and inadequate cleaning of these essential pieces of equipment. The authors recommend that single-use alternatives should not be rejected without consideration.
de Oliveira Batista KC et al. (2015) (16)	Contamination of tourniquets for peripheral intravenous puncture	18 reusable tourniquets	At the laboratory, tourniquets were immersed in single flasks of BHI (brain heart infusion) broth and incubated at 35°C for up to 48 h. Samples with microbial growth were inoculated onto salted mannitol agar and tryptic soy agar with 4% NaCl. They were then incubated at 35°C for up to 48 h. TSA oxa medium was used to isolate and identify oxacillin-resistant strains.	- 11/50 (52,4%) *coagulase-negative Staphyloccocus* - 2/50 (9,5*%*) *S. aureus* - 4/50 (19%) *Rodothorula muciaginosa* - 3/50 (14,3%) *Candida* - 1/50 (4,8%) *Candida parapsilosis*	The results of this study demonstrate that IV tourniquets are widely used by nursing teams and may be contaminated with pathogenic microorganisms that operate as fomites in healthcare environments.
Kane L. et al. (2011) (17)	Phlebotomy tourniquets- vectors for bacterial pathogens	10 tourniquets	Tourniquets were swabbed. Samples were applied to McConkey agar and blood plates, incubated for 48 h, and assessed for bacterial growth.	- 3/10 tourniquet were positive with MRSA Pathogens like c*oagulase negative staphylococci, micrococci, ß- haemolytic streptococci, coliform colonies* were also present in unspecified quantities.	Tourniquets for use in phlebotomy are potential vectors for bacterial pathogens. Clinical facilities should provide disposable tourniquets and promote good infection control practices through education and meticulous hand hygiene to reduce the risk of microbial spread. Staff motivation and compliance with single-use alternatives are significant barriers to reducing cross-infection.

## Discussion

This systematic review of 13 articles summarized the evidence from basic research, epidemiological, and clinical treatment studies of the association between MROs and the use of reusable tourniquets by medical personnel. A previously published research by Pinto et al. primarily focused on the highest rates of multi-resistant organisms in single-patient tourniquets used in intensive care units. In this systematic review, we describe basic research studies on every type of ward in a hospital (13). Evidence suggests that reusable tourniquets may serve as a potential reservoir of bacterial microorganisms in a diverse range of healthcare settings (6, 9). Using disposable tourniquets in hospitals is unpopular; this may be due to the convenience of staff using reusable stasis and a matter of habit. In the study conducted by Grohmann et al., it was shown that there was significantly less bacteria on silicone than on conventional tourniquets. The use of a silicone tourniquet is a more secure, sustainable, and economical alternative to tourniquets made of other materials (1). It is also worth considering if the longer time a patient stays in hospital due to several infections caused by pathogens is better than using disposable tourniquets, which may prevent the spread of infections. Hospital-acquired infections can lead to increased length of stay and higher costs (18). According to US estimates, the cost of a single episode of MRSA bacteraemia is US$26,446, meaning that one episode corresponds to the cost of at least 130,000 disposable tourniquets, considering that a disposable tourniquet costs £ 0.07, according to UK cost estimates quoted by Leitch et al. (11, 19). It is also interesting to explore if pathogens from tourniquets may contribute to infections within the IV. Disposable tourniquets are ideal, but as long as reusable tourniquets are used for various reasons, adequate infection prevention is required. Kerstain et al. recommended the use of disposable tourniquets, claiming that, taking into account the cost of hospital-acquired infections and the length of time a patient stays in the hospital due to infection, it is more cost-effective to reduce infection transmission to patients by the use of disposable tourniquets (20). Contamination of tourniquets depends on the hospital as a whole. Poor hygiene in combination with the careless use of tourniquets makes them a source of hospital-acquired infections. (2). Kerstain et al. found that disposable tourniquets are favored by patients and phlebotomists for their convenience and practicality (20). Using single-use devices for blood sampling and drawing is proposed by the *WHO Guidelines on Drawing Blood: Best Practices in Phlebotomy* (7). Compression tourniquets, as nursing medical devices, should be stored and managed with greater care to limit negative effects on patients. Steps to be taken should include adopting protocols, educating staff, and providing stasis disinfection between uses (21). A study by Schauer et al. demonstrated that the quantitative risk of reusable tourniquets appears to be low using standard infection control practices (22). Regular supervision and rules for sterilization or disinfection of tourniquets are advised in the Infection Control program to reduce hospital-acquired infections. (23).

National guidance on the use and cleaning of venipuncture tourniquets is recommended (24).

### Limitations

Some important scientific research was left out of the search and selection process due to the nature of the publication date (research since 1986) and language (English). Tests have been carried out in different regions, so the cultural aspect and general hospital policy environment in the country should be taken into account. It is necessary to mention the methodological limitations of the systematic review, including inherent biases that include selection, different quantitative sampling based on unspecified reusable tourniquet methods, various locations for collecting materials, miscellaneous infection control policies in medical facilities, and effective enforcement of glove and hand hygiene. Furthermore, internal validity was achieved in this study as there was no bias in the study design, analysis, and how the study was conducted. To ensure the internal validity of the study, the selection of tourniquets was randomized and we followed specific procedures during the study. There were limitations in that generalization to external validation was not possible.

## Conclusion

Patient well-being may be at risk due to the high level of proportional observation based on the number of sampled contamination of reusable tourniquets. The micro-organisms responsible for this contamination belong to different species, the most common being the genus *Staphylococcus.* For this reason, we suggest the use of disposable tourniquets.

### Implications for practice

The review identified weaknesses in the use of reusable tourniquets in medical settings. Safely collecting blood using tourniquets that will not threaten the patient’s safety should be a priority even if it is challenging for medical facilities, which should focus on the adoption of clear rules of conduct. In practice, more emphasis should be placed on monitoring the epidemiological status of reusable tourniquets in medical facilities.

## Data availability statement

The original contributions presented in the study are included in the article/supplementary material, further inquiries can be directed to the corresponding author.

## Author contributions

JS: Conceptualization, Data curation, Formal analysis, Software, Writing – original draft. MM: Conceptualization, Data curation, Writing – original draft. WM-D: Conceptualization, Methodology, Visualization, Writing – review & editing.

## Footnotes

^1^ https://jbi-global-wiki.refined.site/space/MANUAL

## Author’s Note

The article was written as part of the Journal Club Nursing project at the Medical University of Gdańsk.
